# The role of hydro‐environmental factors in Mayfly (Ephemeroptera, Insecta) community structure: Identifying threshold responses

**DOI:** 10.1002/ece3.6333

**Published:** 2020-07-08

**Authors:** Pfananani A. Ramulifho, Stefan H. Foord, Nick A. Rivers‐Moore

**Affiliations:** ^1^ SARChI‐Chair in Biodiversity Value and Change University of Venda Thohoyandou South Africa; ^2^ Department of Zoology and Centre for Invasion Biology University of Venda Thohoyandou South Africa; ^3^ Centre for Water Resources Research University of KwaZulu‐Natal Scottsville South Africa

**Keywords:** global change, instream communities, Luvuvhu River catchment, stream flow, thermal change, TITAN, water temperature

## Abstract

Freshwater organisms are threatened by changes in stream flow and water temperature regimes due to global climate change and anthropogenic activities. Threats include the disappearance of narrow‐tolerance species and loss of favorable thermal conditions for cold‐adapted organisms. Mayflies are an abundant and diverse indicator of river health that performs important functional roles. The relative importance of key hydro‐environmental factors such as water temperature and flow volumes in structuring these communities has rarely been explored in the tropical regions of Africa. Here, we investigate the response of mayfly species diversity to these factors in the Luvuvhu catchment, a strategic water source area in the arid northeastern region of South Africa. Mayfly larvae were sampled monthly in stones‐in‐current biotopes across 23 sites over a one‐year period. The relationship between these environmental drivers and mayfly diversity was modeled using linear mixed effects models (LMMs) and a model‐based multivariate approach. Threshold Indicator Taxa Analysis (TITAN) was used to model the response of mayfly species to important gradients and identify thresholds of change. Site‐specific characteristic were the most important predictor of mayfly diversity, and there was considerable variation over time, with mayfly diversity peaking during winter. Along this, gradient temperature was the best predictor of assemblage structure, with five out of six reliable indicator species being cold‐adapted, and a community threshold response at 19°C. Results support laboratory‐based thresholds of temperature for mayfly species survival and development, extending empirical evidence to include field‐based observations. Increased global (climate change) and local (riparian vegetation removal, impoundments) changes are predicted to have negative impacts on mayfly diversity and ultimately on ecosystem function.

## Graphical abstract

Mayflies, one of the most abundant and diverse benthic invertebrate orders, perform important functional roles under the influence of temperature and flow signatures in the Luvuvhu catchment. Mayfly are thermal indicators, decreasing with increasing temperatures, at a temperature threshold of 19°C.

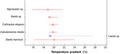

## 1 INTRODUCTION

Freshwater ecosystems cover less than 1% of the earth's surface, but provide a home to 10% of all known animal species, 60% of which are aquatic insects (Dijkstra, Monaghan, & Pauls, 2014). Stream flow and water temperature are important regulators of distribution and survival of these aquatic macroinvertebrate species. Flow and temperature regimes are increasingly threatened by anthropogenic activities which include habitat fragmentation, removal of riparian zones, eutrophication, abstraction, pollution, as well as global climate change and its associated increase in drought and flood frequency (Dallas & Rivers‐Moore, 2014; Olden & Naiman, 2010). Rivers, in particular, hold the highest proportion of benthic organisms threatened by climate change, influencing and altering assemblage structure, resulting in a loss of species diversity, and ultimately altering ecosystem function (Bunn & Arthington, 2002; Dallas & Rivers‐Moore, 2014).

Mayflies (Ephemeroptera) are a major component of these aquatic macroinvertebrate communities, with aquatic nymphs that are extremely diverse in shape and structure, reflecting their highly diverse habitats, locomotion, and feeding behavior (Baptista et al., 2006; Sartori & Brittain, 2015). They are ubiquitous in every kind of freshwater ecosystem (Alhejoj, Elias, & Klaus, 2014; Buss & Salles, 2007), representing the fourth largest purely aquatic invertebrate order in streams and rivers (Dijkstra et al., 2014), while decreasing in lakes and ponds (Barber‐James, Gattolliat, Sartori, & Hubbard, 2008). Taxonomically, mayflies are relatively well studied (Dijkstra et al., 2014), with their entirely aquatic nymphs, representing the longest developmental stage in the life cycle of these organisms (Barber‐James, 2016). Mayflies are distributed worldwide with over 3,000 species, in more than 400 genera and 42 families (Barber‐James et al., 2008).

Mayfly distribution is largely related to substrate type, water velocity, depth, turbulence, temperature, and hydraulic parameters (Buss & Salles, 2007; Gustafson, 2008; Vilenica, Andreja, Michel, & Mihaljevi, 2018), which are in turn influenced by a river's thermal and flow signature (Rivers‐Moore, Dallas, & Morris, 2013). Water temperature and flow are major drivers of river ecosystems (Dallas & Rivers‐Moore, 2014; Poff & Zimmerman, 2010) and mayfly assemblage structure in running waters (Gustafson, 2008; Nelson & Lieberman, 2002; Pardo, Campbell, & Brittain, 1998; Vilenica, Mi, Sartori, Ku, & Mihaljevi, 2017). This is because mayfly are ectothermic, and their fitness and physiology depends on flow and temperature for both their dispersal and development. (Chessman, 2012; Hawkins & Hogue, 1997). Oxygen availability, stream size, competition for food and space, resource availability, water chemistry, and light provide finer scale filters of community structures (Brooks, Haeusler, Reinfelds, & Williams, 2005; Christidis et al., 2017; Finn & Poff, 2005; Svitok, 2006), while human activities now impact mayfly community structures at larger scales (Klonowska‐Olejnik & Skalski, 2014) through flow regulation (Bunn & Arthington, 2002), the removal of riparian forest (Siegloch, Suriano, Spies, & Fonseca‐Gessner, 2014), and bank degradation for agricultural activities amongst others (Allan, 2004).

The occurrence and distribution of mayfly communities have been found to decrease from ecologically pristine to moderately disturbed habitats (Bauernfeind & Moog, 2000). Diversity also generally decreases with an increase in altitude (Jacobus, Macadam, & Sartori, 2019). In general, mayflies are especially diversified in temperate and tropical environments (Sartori & Brittain, 2015) and decreases toward the poles due to their ecological (suitable habitat) requirements (Jacobus et al., 2019). Until 2015, the Palearctic region was characterized as having the highest mayfly diversity, with 790 described mayfly species by 2005 (Barber‐James et al., 2008). Currently, the Neotropics are recognized as the most diverse region with 900 described species, followed by the Palearctic (830), Nearctic and Oriental (610 and 620, respectively), Afrotropical (440) Australasian (250), and Pacific (48) (Jacobus et al., 2019).

Mayfly species play a fundamental functional role in stream ecosystems as consumers (filterers, shredders and collectors) and prey at intermediate trophic levels acting as conduits of bottom‐up and top‐down components (Baptista et al., 2006; Wallace & Webster, 1996). Mayfly community and functional feeding groups change as streams grow wider downstream from the source to the mouth of a river (Vannote, Minshall, Cummins, Sedell, & Cushing, 1980), a pattern widely applicable to temperate streams (Masese et al., 2014). Predictable change in aquatic organisms downstream is difficult to apply in many tropical streams, with increasing evidence that related species occurring in tropical areas and other regions do not share the same diets (Masese et al., 2014). Even within regions, some taxa can shift their feeding in response to changes in land use and riparian conditions (Masese et al., 2014).

The frequency and abundance of more sensitive mayfly taxa decline along stressful environmental gradients over space or time, resulting in changing species composition and dominance (Costas, Pardo, Méndez‐Fernández, Martínez‐Madrid, & Rodríguez, 2018; Klonowska‐Olejnik & Skalski, 2014). Knowledge of mayfly community composition, seasonal dynamics, distribution, and their narrow habitat sensitivity extends their utility value beyond just indicators and surrogates of habitat change (Bauernfeind & Moog, 2000; Vilenica, Ivković, Sartori, & Mihaljević, 2017) to being agents for adaptive and holistic conservation planning of freshwater resources (Ramulifho, Rivers‐Moore, Dallas, & Foord, 2018). The choice of mayflies in river monitoring programs lies in the low cost of sampling associated with their collection and their high sensitivity level to water quality parameters (Snyder, Hitt, Smith, & Daily, 2014).

The literature on the relationships between hydro‐environmental variables and assemblage structure of aquatic organisms is limited for Afrotropical streams, particularly for the northern regions of South Africa, where it is largely restricted to rapid biological assessments (Foord & Fouché, 2016). Here, we explore the relationship between stream flow, water temperature, and other important covariates that drive mayfly assemblage structure in the Luvuvhu catchment. We aim to (a) describe mayfly community composition in “stones‐in‐current” biotopes for five major tributaries of the catchment, (b) explore the role of hydro‐environmental variables, particularly flow and temperature, in explaining mayfly diversity, and (c) identify threshold responses to key drivers. We hypothesized that water temperature is the main driver of the mayfly richness because of their high sensitivity rate to change in temperature gradient.

## 2 METHODS

### 2.1 Study area

**FIGURE 1:**
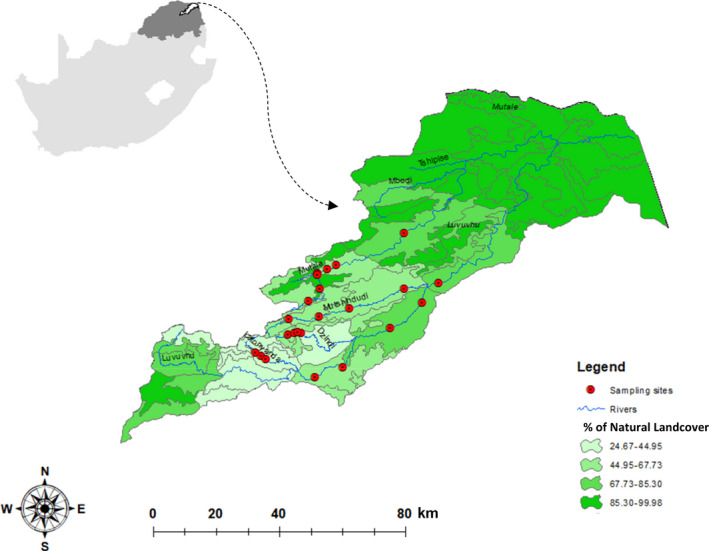
Location of sampling sites and the percentage of natural land cover in the Luvuvhu catchment, Limpopo province, RSA. Insert shows location of study region in the northeastern of both Limpopo and South Africa

The Luvuvhu River catchment is a strategic water source area (Nel, Colvin, Le Maitre, Smith, & Haines, 2013) in the northeastern arid region of South Africa (Figure 1). It covers an approximate area of 5,940 km^2^, with a mean annual precipitation (MAP) of 608 mm, mean annual runoff (MAR) of 519 million m^3^ (ranging from 85 to 1,900 million m^3^), and an elevation range between 232 and 1,587 m asl (DWAF, 2012). Rivers of this catchment have shown a substantial decrease (>53%) in stream flow volume over the last 80 years (Odiyo, Makungo, & Nkuna, 2015). Kleynhans (1996) classified streams in the Luvuvhu River as fairly natural, but recent agricultural intensification and the expansion of human settlements have had substantial impacts on instream biota (Foord & Fouché, 2016), and the flow regime has consequently been altered considerably (Ramulifho, Ndou, Thifhulufhelwi, & Dalu, 2019).

### 2.2 Ephemeroptera sampling

Twenty‐three sites (Figure 1; Table S1) across six perennial streams in the Luvuvhu catchment were sampled based on the presence of a hydraulic biotope of shallow running water with cobbles. Only cobbles with diameter between 10 and 30 cm were selected to ensure that only one biotope type “stones‐in‐current” was sampled (Chutter, 1972). Mayfly diversity peaks in stony substrates (Christidis et al., 2017; Vilenica, Mi, et al., 2017) and is largely absent at sites lower than 450 m asl which are dominated by sandy substrates and have almost no “stones‐in‐current” biotopes in this catchment. The “stones‐in‐current” biotope was also absent at higher elevations (>1,000 m). Initially, fourteen sites were sampled monthly to complete an annual cycle (December 2016–January 2018), while nine additional sites were included for the period between April 2017 and January 2018 to allow for increased representation of environmental gradients. During each of the monthly surveys, six stones in each site were rinsed and brushed to dislodge organisms and trapped downstream in a sampling net (30 × 30 cm; 250 µm mesh). Contents of the net were emptied into a sample bottle and sorted in the laboratory. All specimens were identified to the highest taxonomic level possible using De Moor, Day, and De Moor (2003). Identifications were subsequently confirmed by mayfly taxonomists at the Albany Museum, South Africa. The organisms were preserved in 70% alcohol and deposited in a reference collection of benthic macroinvertebrates, at the University of Venda, South Africa.

### 2.3 Environmental variables

Instantaneous measurements for flow depth and flow velocity above each of the six stones were recorded using a Flow Globe FP101 (Global Water), while instantaneous readings for pH, water temperature (WT), total dissolved solids (TDS), and electrical conductivity (EC) were measured once at each site using portable multi‐parameter water transmitters “Crison pH/mV” (Table S1). The channel width at each sampling site was measured in meters. Dimensions (height, length and width) of all stones sampled in the first month were measured in centimeters and surface area estimated by covering them with metal foil, which was later weighed (Cooper & Testa, 2001). The surface area of subsequent stones was calculated using a regression equation with stone volume as the dependent variable (Cooper & Testa, 2001). Land cover characterization was obtained by on‐screen digitizing from multi‐seasonal, 30 meter resolution Landsat 8 satellite imagery, acquired between April 2013 and June 2014 (Geoterraimage, 2015). The 72 classes land cover data were reclassified into two classes (natural vs. non‐natural) by means of percentage in the 30 × 30 m resolution image at the quinary catchment level using 1:50,000 mapping and modeling scale.

### 2.4 Statistical analysis

All statistical analyses were done in R version 3.6.1 (R Core Team, 2017). Abundance data for mayflies were pooled across six stones within a site, as counts of mayflies on individual stones were zero inflated. These and their associated predictor variables that were included in the analysis and resulted in 14 sites sampled for 12 months, and nine sites sampled for 10 months equaling a total of 258 samples. Colinearity between environmental variables was explored using principal component analysis (PCA) and Pearson's correlation coefficients. The first and second principal components of the PCA explained 30% and 19% of the total variance, respectively (Figure S1). As expected, sites at higher elevations were narrower, colder, had lower conductivity, and had more acidic water (Figure S1), also, sites with higher flow had larger rocks. Correlated variables were not included in the same model (Figure S1).

Sample coverage was calculated for observed community richness using the function “iNEXT” in the iNEXT package (Chao & Jost, 2012; Sieh, Ma, & Chao, 2014). Observed species richness had a normal probability distribution, and we therefore used linear mixed effects models (LMM) with the function “lmer” and an identity link function in the lme4 package to model richness (Bates et al., 2014). Model residuals were also inspected for normality and heteroscedasticity. Sites were included as a random factor to account for temporal pseudoreplication. Survey dates and river were included as a categorical variables, while quadratic terms for numerical predictors were included as fixed effects. Prior weights for observed richness were added using the coverage for each sample. Models were compared using the Akaike's information criterion (Li et al., 2018) to identify those models minimizing the loss of information (Barton, 2018). Models with ΔAIC ≤ 2 from the best model were considered equivalent (Burnham & Anderson, 2002). Marginal *R* ^2^_m_ (variation explained by fixed effects only) and conditional *R* ^2^_c_ (variation explained by fixed and random effects) were calculated for the best random intercept model (Nakagawa & Schielzeth, 2013).

We fitted multivariate generalized linear models (GLMs) to abundance data of mayfly in the R package “mvabund” (Wang, Neuman, Wright, & Warton, 2012) using the functions “manyglm” and “ANOVA.manyglm.” This model‐based approach is superior to a distance‐based methods, as multivariate GLMs account for confounding mean–variance relationships that commonly arise in abundance data which contain many zeros (Warton, Thibaut, & Wang, 2017). We used this method to explore the importance of predictor variables in explaining mayfly assemblage structure. Conditional effects were calculated by summing the likelihood ratio statistics for each taxon, yielding a community‐level measure for each of the predictors. The likelihood ratio statistic was calculated for each species as a measure of effect for each predictor and summed. Correlation between species was accounted for by using the PIT‐residual bootstrap method to derive p‐values by resampling 999 rows of the data set (Warton et al., 2017). Marginal explanatory power was explored by including the predictors individually into the model and calculating the deviance explained. Colinearity was calculated using the variance inflation factor (VIF). Only predictors with a VIF < 3 were retained. Model assumptions were explored by visually examining the plot of residuals for normality, constant mean–variance relationship, and independence.

Threshold Indicator Taxa Analysis (TITAN) from the “TITAN2” package (Baker & King, 2010) was used to identify the change‐point response of mayfly communities to the predictor variable that explained most of the variation in assemblage structure. The TITAN method uses the standardized *z*‐scores obtained from indicator species analysis (Indicator Value) to detect the taxon‐specific change points and the response direction of a taxon along an environmental gradient (Baker & King, 2010; Costas et al., 2018). Standardized taxa responses increasing at the change point (*z*+) are distinguished from those decreasing (*z*) and those showing no response (Baker & King, 2010). By means of bootstrapping, TITAN estimates indicator reliability and the proportion of times that a taxon is given the same classification in each bootstrap replicate as in the observed data set, as well as uncertainty around the location of individual taxa and community change points (Baker & King, 2010).

## 3 RESULTS

A total of 11,041 mayfly larvae, comprising 19 species in 16 genera and six families, were recorded (Table S2). Species richness varied between three and 15 species per site (Table S2). *Baetis*, which had several undefined species, and *Dabulamanzia media* were the most abundant genus and species, respectively, while *Afroptilum sudafricanum* was the rarest species with only one individual sampled. Flow depth varied from 0 to 39 cm and averaged at 15 cm.

**FIGURE 2:**
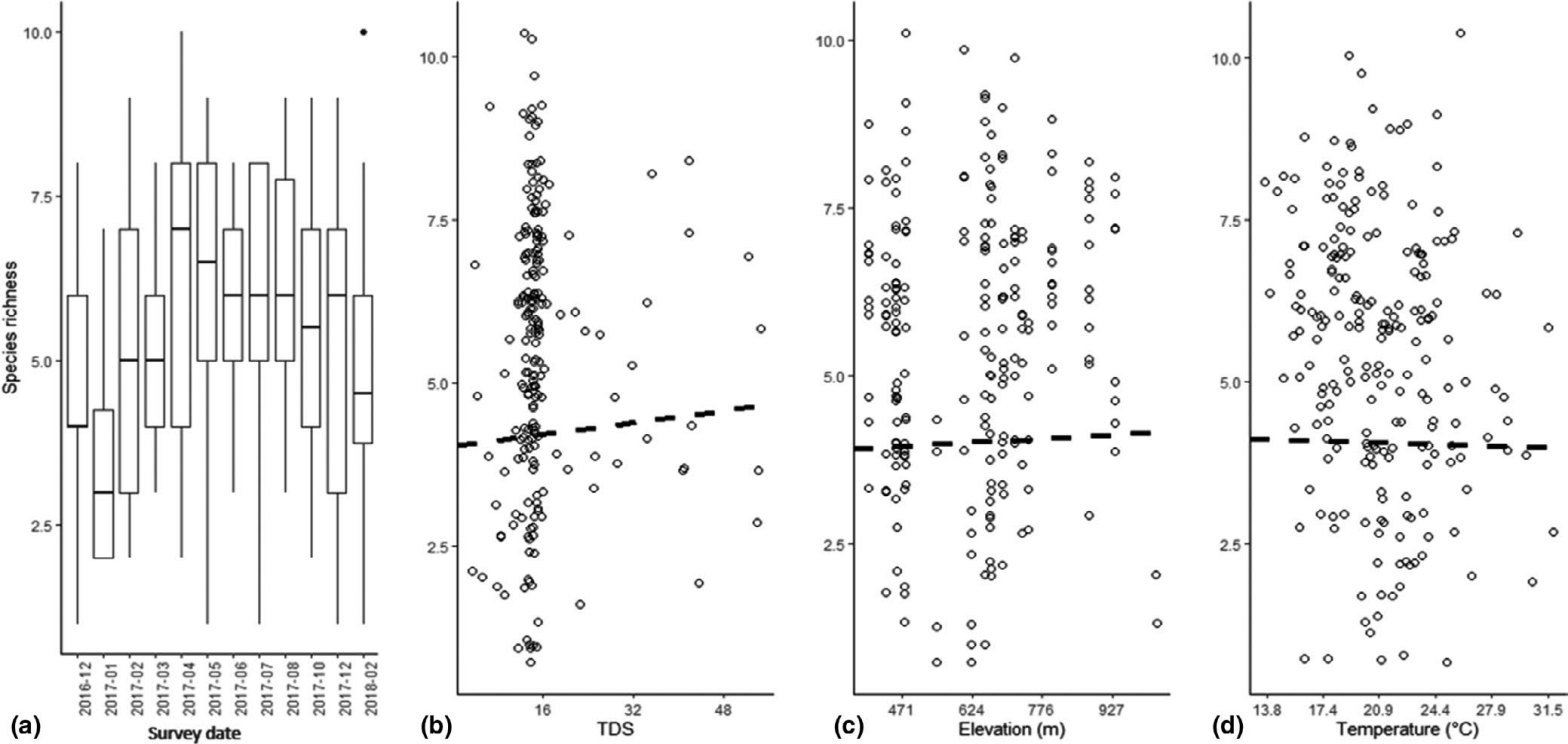
Relationship between richness and (a) survey date, (b) TDS, (c) elevation, and (d) temperature. Dashed line indicates fitted regression between two variables

A mean number of five species were observed at a site during each survey and sample coverage averaged at 0.96. We consider sampling at a site to be representative of the community as the species sampled constitute 96% of the abundance of the whole assemblage at a site. The best model for richness included survey date as the only fixed variable (Table 1) and richness varied considerably between surveys, peaking during winter months (Figure 2a). The next three models included either TDS, elevation, or temperature as fixed variables together with survey date (Table 1). Richness increased with increases in both elevation and TDS, while it decreased at higher temperatures (Figure 2). These relationships were however weak. Marginal and conditional variations explained were similar for all models, 14%–15% and 52%–53%, respectively, which suggests that there were site‐specific characteristics, not measured here, that had a considerable impact on variation in richness.

**TABLE 1:** Summary of models predicting species richness

Model	Log likelihood	*df*	AIC	ΔAIC	*R* ^2^_m_	*R* ^2^_c_
Richness = survey date	−425.04	14	878.08	0	.15	.52
Richness = survey date + TDS	−424.84	15	879.68	1.6	.15	.52
Richness = survey date + elevation	−425.02	15	880.03	1.95	.14	.53
Richness = survey date + temperature	−425.03	15	880.07	1.99	.15	.52

Both conditional and marginal effects suggest that sites were the most influential predictor of assemblage structure (Table 2), further emphasizing the importance of site‐specific characteristics in structuring mayfly assemblages. However, temperature explained the largest amount of conditional variation and followed sites in the total amount of deviance explained, while the remaining predictors had no significant conditional effects or much less marginal effects than sites and temperature.

**TABLE 2:** Conditional (Likelihood ratio and its significance) and marginal effects (Deviance) of predictors for mayfly assemblage

Predictor	Conditional effects Likelihood ratio (LR)	Marginal effects Deviance
Site		
Dzindi above Waterfall	30.58*	384.5
Dzindi below Waterfall	47.73*	
Hasani	54.23*	
Lutanandwa	23.23	
Lutanandwa Bridge	30.46	
Lwamondo	40.16*	
Malavuwe	40.2*	
Mapate	28.2*	
Midmutale	43.87*	
Mutale Bridge	61.26*	
Nandoni	45.48*	
Phiphidi	40.21*	
Tea Estate	59.27*	
Thathewaterfall	37.13*	
Tshanzhe	58.79*	
Tshikonelo	21.95	
Tshino	11.51	
Tshirovha	75.4*	
Tshirovha Forest	89.06*	
Tshirovha potholes	31.97*	
Tshivhulani	43.12*	
Upper Lutanandwa	45.46*	
Width	11.49	1,215.5
Temperature	172.57*	1,167
pH	33.47	1,268
Conductivity	26.17	1,290
Flow	14.11	1,260
Depth	24.58	1,276
Rock Size	39.03	1,258

Note

Significance: *p < .05, **p < .01, and ***p < .001.

**FIGURE 3:**
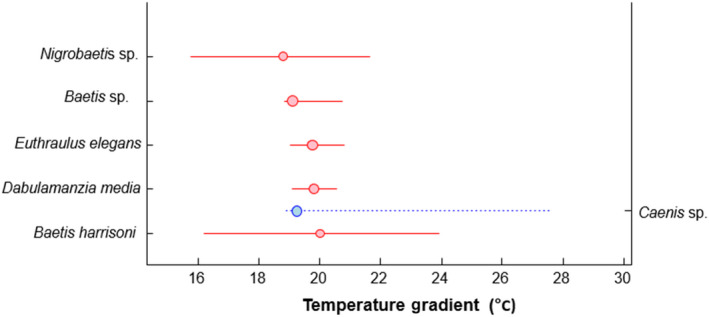
Threshold Indicator Taxa Analysis (TITAN) of mayfly species community response to a water temperature gradient in the Luvuvhu catchment (°C). Red symbols correspond to species that respond negatively (*z*−) to temperature increases, and blue symbols are species that has a positive (*z*+) response. Symbols are in size proportional to *z* scores. Horizontal lines show 5th and 95th percentiles among 500 bootstrap replicates

**FIGURE 4:**
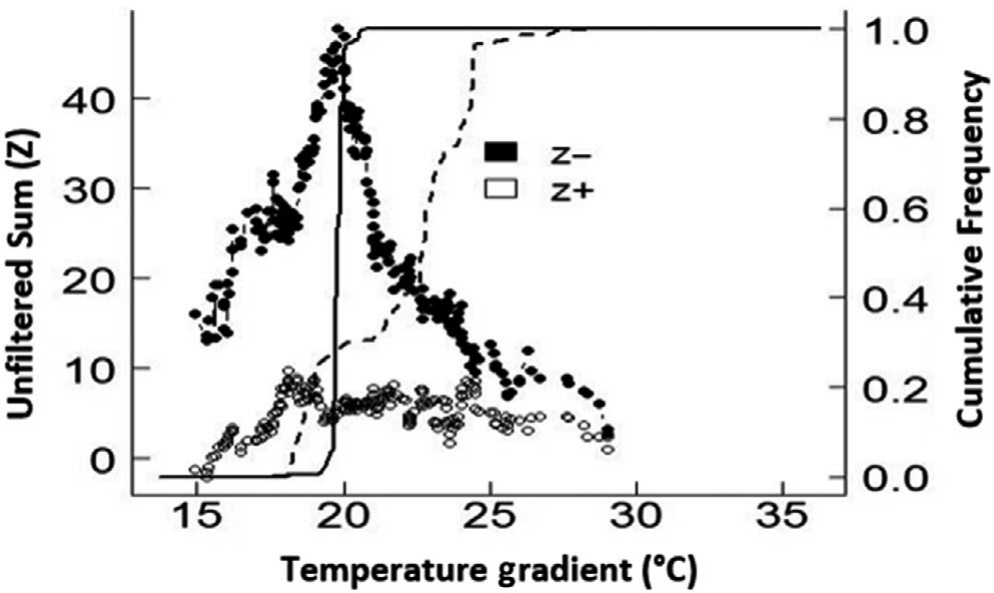
Change‐point analysis of mayfly species community in response to temperature. Peaks in sum (*z*−) and sum (*z*+) correspond to locations along the gradient where there are synchronous declines (*z*−) and increases (*z*+) in mayfly species abundance. Solid and dashed lines represent the cumulative frequency distribution of change points among 100 bootstrap replicates for sum (*z*−) and sum (*z*+), respectively

Only the gradient change point of temperature was modeled as it was the only continuous predictor which had a significant effect on assemblage structure. Six mayfly species were identified as indicators of change along the temperature gradient (Figure 3). The abundance of five of these taxa declined in response to temperature with threshold temperature of 19°C (Figure 4). These species were *Nigrobaetis* sp., *Baetis* sp., *Euthraulus elegans*, *Dabulamanzia media*, and *Baetis harrisoni*. *Nigrobaetis* sp. and *Baetis harrisoni* all of which were thermophobic. Only one species, *Caenis* sp., had a thermophilic response.

## 4 DISCUSSION

### 4.1 Drivers of assemblage structures

Similar to other studies (Brooks et al., 2005; Vilenica et al., 2018), results suggest that site‐specific characteristics and seasonal change are important determinants of mayfly diversity. Oxygen content (Vilenica, Mi, et al., 2017), food availability (Svitok, 2006), percentage of native angiosperms and mosses, and substrate roughness as a refuge from predators and hydraulic disturbance (Brooks et al., 2005; Vilenica et al., 2018) might be some of the important site‐specific characteristics that were not measured. Although variables measured had a weak effect on species richness at the community level, temperature in particular, consistently explained significant amounts of variation and threshold responses of mayfly species suggest that 83% are thermophobic, decreasing with increases in temperature.

Gustafson (2008) provided evidence for the importance of temperature in structuring mayfly communities. Though literature on life histories patterns of Afrotropical mayfly diversity are scarce (Barber‐James et al., 2008), evolutionary history suggests that many mayfly species has always depended on temperature for their organismic, population, and species level structures (Brittain, 2008; Edmunds, 1972; Wolda & Flowers, 1984). A number of studies have indicated water temperature as the most important environmental driver of assemblage structure of mayfly communities (Gustafson, 2008; Haidekker & Hering, 2008; Vilenica, Mi, et al., 2017). This is because temperature is closely related to species traits such as embryonic development, nymphal growth, emergence, metabolism, and survivorship of many taxa (Dallas & Rivers‐Moore, 2012, 2018; Haidekker & Hering, 2008; Vilenica, Mi, et al., 2017). However, the high correlation between mayfly richness and winter conditions in this study is uncommon for this aquatic group. Warm adapted species, like many mayfly, develop faster and are active at higher temperatures as opposed to their winter quiescence (Haidekker & Hering, 2008). Some studies have also observed no mayfly species activities and ceased growth during the freezing winter temperatures in regions at higher latitudes (Dallas, 2016; Rader & Ward, 1990).

The level of pH also had a considerable effect on mayfly assemblages. This water parameter regulates important physiological functions of mayflies, including the exchange of ions with the water and respiration (Kubendran, Selvakumar, Sidhu, Nair, & Krishnan, 2017; Svitok, 2006; Vilenica, Ivković, et al., 2017) which otherwise is impossible to operate normally under extreme pH values (>8.5 and <6.5) (Klonowska‐Olejnik & Skalski, 2014). In this study, water tends to get more acidic at higher elevation sites because of less anthropogenic impacts that include sewage discharge, car washing, body and clothes washing, animal grazing, and subsistence farming which contribute to the rising level of pH (Okonkwo & Mothiba, 2005). An increasing pH caused by detergents and bathing soaps has been a concern in large areas of the Luvuvhu catchment (Kleynhans, 1996; Traoré et al., 2016).

Stream flow was another important environmental driver of mayfly species abundance or occurrence. A study by Klonowska‐Olejnik and Skalski (2014) shows that stream regulation and organic pollution have influence on mayfly community structure. This is because some species are linked to frequent flooding and increased discharge due to heavy rainfall, while some prefer slow flowing water (Sartori & Brittain, 2015; Siegloch et al., 2014). This is not surprising given the influence of stream regulation on stream flow which causes numerous changes (Bunn & Arthington, 2002), affecting hydraulic patterns (velocity and depth) and variation on mayfly assemblage structures (Brooks et al., 2005; Klonowska‐Olejnik & Skalski, 2014; Salmaso et al., 2018). With increasing stream flow variability and declining stream flow volumes in the Luvuvhu catchment due to increasing water abstraction and climate variability (Kleynhans, 1996; MacFadyen, Zambatis, Astrid, Van, & Cang, 2018; Ramulifho et al., 2019), this may decrease abundance or diversity of mayfly as well their ecosystem services (Boyero et al., 2011).

The absence of percentage natural cover contrasts with other studies in tropical regions, for example, in southeastern Brazil, Siegloch et al. (2014) showed a 57% reduction in mayfly richness in streams with decreased natural vegetation cover. Pond (2010) also found that mean mayfly richness and relative abundance were significantly higher in naturally vegetated catchments. Naturalness in this instance was determined by the degree of transformation of a catchment. Large scale commercial timber production was considered least natural in this context. Areas with high natural vegetation cover occur in lower parts of Luvuvhu catchment. However, riparian zones in these landscapes are largely intact and might suggest richness was not negatively affected. Classifying vegetation zones into natural and transformed using remote sensing techniques might therefore underestimate the positive local effects of intact riparian zones.

### 4.2 Indicator taxa

Identifying reliable indicator taxa and their responses to changing environmental gradients is of major concern for the development of management tools for freshwater ecosystems (Costas et al., 2018). In this study, temperature was the most consistent variable determining mayfly assemblage structure after survey date hence its threshold examination in relation to mayfly response. The majority of mayfly species (83%) responded negatively to increased water temperatures, while only one species increased. A study by Rivers‐Moore, Dallas, and Ross‐Gillespie (2013) associated rising temperature to loss of favorable thermal habitat for cold‐adapted mayflies. Thermally vulnerable and thermorphobic species will need to extend their distribution to suitable and accessible habitats at higher altitudes as the climate changes (e.g., Bush, Nipperess, Turak, & Hughes, 2012; Filipe, Lawrence, & Bonada, 2012; Walther, 2010). Laboratory assays of *L. penicillata*, a mayfly species of conservation importance, found that the chronic thermal stress threshold ranged from 19.2 to 20.7°C depending on season (Dallas & Rivers‐Moore, 2018). This coincides with our field‐based observation of 19°C and provides compelling support for the importance of these temperatures to both individuals and community‐level responses. A field‐based observation by Vilenica, Ivković, et al. (2017) also identified threshold responses at 18°C for some mayfly species inhabiting the mountainous rivers in the Mediterranean region. The survival of many species under climate change will depend on their ability to disperse and colonize new favorable sites though this is limited by the recurrent fragmentation of river networks (Bruno et al., 2019).

## 5 CONCLUSION

The significant impacts of climate change and instream impoundments on stream flow and water temperature regimes will undoubtedly lead to significant changes in mayfly communities (Brittain, 2008; Rivers‐Moore, Dallas, & Ross‐Gillespie, 2013; Sartori & Brittain, 2015). Climate change will represent a major driver of future biodiversity loss in stream and rivers, because these environments will be mostly inhabited by cold stenothermal organisms (Fenoglio, Bo, Cucco, Mercalli, & Malacarne, 2010). Global warming will enhance extinction rates of native species, because of cold water habitat reduction, dissolved oxygen depletion, changes in the ecological functioning, and introduction of allochthonous invaders (Boyero et al., 2011; Fenoglio et al., 2010; Sartori & Brittain, 2015). However, warming of stream water due to the effects of global climate change can be reduced by maintaining the natural instream habitat and riparian zones and limiting hydrological abstraction to increase resilience in freshwater ecosystem (Dallas & Rivers‐Moore, 2018). In this study, we have provided evidence for the importance of thermal regimes in structuring mayfly assemblages with real implications for mayfly diversity under global change scenarios that include climate and land use. Since the presence or absence of certain mayflies is strongly influenced by temperature, as indicators, mayflies can help to establish thresholds levels of unacceptable thermal degradation in freshwater ecosystems.

## CONFLICT OF INTEREST

The authors declare that there are no conflicts of interests.

## AUTHOR CONTRIBUTIONS

**Pfananani A. Ramulifho:** Conceptualization (equal); Data curation (equal); Formal analysis (equal); Methodology (equal); Project administration (equal); Writing‐original draft (equal); Writing‐review & editing (equal). **Stefan H. Foord:** Conceptualization (equal); Funding acquisition (equal); Software (equal); Supervision (equal); Validation (equal); Visualization (equal). **Nick A. Rivers‐Moore:** Conceptualization (equal); Resources (equal); Supervision (equal); Validation (equal); Writing‐review & editing (equal).

## Supporting information

Supplementary Material

## Data Availability

Raw data associated with this paper are available at https://doi.org/10.6084/m9.figshare.12124158
