# Supplementary material for: The role of hydro‐environmental factors in Mayfly (Ephemeroptera, Insecta) community structure: Identifying threshold responses
Source: Ecol Evol. 2020 Jul 8;10(14):6919–28. doi: 10.1002/ece3.6333 (PMC7391557; doi:10.1002/ece3.6333)
Supplement: Supplementary file 1 — Supplementary Material [file ECE3-10-6919-s001.docx]

Table S1: Sites, mayfly abundance, richness, vegetation cover and average scores of environmental variables measured at sampling sites in the Luvuvhu catchment between December 2016 and February 2018.

| **Site Name** | **Samples** | **Altitude** | **Temp** | **Flow** | **Depth** | **Width** | **TDS** | **pH** | **Conductivity** | **Natural Cover %** | **Total Mayfly abundance recorded** | **Mayfly Species Richness recorded** |
| --- | --- | --- | --- | --- | --- | --- | --- | --- | --- | --- | --- | --- |
| Thathe waterfall | 12 | 1022.94 | 19.21 ±3.18 | 1.30 ±0.90 | 10.81 ±4.77 | 03.34 ±1.70 | 11.88 ±4.50 | 06.83 ±0.95 | 18.37  ±7.50 | 91.44 | 6 | 3 |
| Tshirovha | 12 | 665.26 | 20.22 ±3.38 | 2.05 ±1.20 | 14.75 ±5.96 | 14.08 ±7.33 | 13.02 ±2.91 | 07.23 ±0.82 | 18.15  ±6.71 | 63.21 | 337 | 13 |
| Midmutale | 12 | 667.52 | 22.58 ±3.39 | 1.83 ±1.18 | 20.73 ±10.30 | 12.26 ±1.23 | 13.81 ±3.17 | 07.05 ±0.27 | 21.29  ±4.67 | 63.21 | 314 | 12 |
| Mutale Bridge | 12 | 651.96 | 20.48 ±3.65 | 2.61 ±1.56 | 15.86 ±7.08 | 18.25 ±5.32 | 13.35 ±3.51 | 06.46 ±0.30 | 21.00  ±5.47 | 63.21 | 361 | 11 |
| Tea Estate | 12 | 876.59 | 19.18 ±3.06 | 2.25 ±1.25 | 16.04 ±5.85 | 08.58 ±1.32 | 12.51 ±3.53 | 06.90 ±0.59 | 17.53  ±7.69 | 51.33 | 911 | 11 |
| Tshivhulani | 12 | 475.71 | 21.39  ±3.08 | 1.27 ±0.58 | 15.33 ±6.67 | 09.87 ±0.81 | 19.97 ±10.26 | 07.69 ±0.20 | 27.63  ±11.33 | 60.73 | 209 | 12 |
| Malavuwe | 12 | 484.24 | 21.71 ±3.44 | 1.58 ±1.02 | 12.31 ±2.92 | 19.66 ±4.87 | 15.93 ±5.46 | 07.61 ±0.34 | 25.38  ±8.63 | 60.73 | 822 | 12 |
| Lwamondo | 12 | 796.01 | 17.96 ±1.47 | 1.93 ±1.00 | 14.90 ±7.09 | 07.73 ±1.11 | 13.49 ±3.33 | 07.22 ±0.43 | 20.88  ±5.12 | 51.12 | 1040 | 11 |
| Mapate | 12 | 688.13 | 20.31 ±1.81 | 2.12 ±0.91 | 15.91 ±7.01 | 09.82 ±1.32 | 13.26 ±3.71 | 07.58 ±0.37 | 19.73  ±6.66 | 30.64 | 634 | 11 |
| Lutananda | 12 | 716.14 | 20.08 ±2.95 | 1.86 ±0.87 | 14.59 ±5.86 | 06.00 ±1.06 | 15.93 ±6.01 | 07.33 ±0.45 | 2.51  ±8.13 | 27.59 | 919 | 13 |
| Tshino | 12 | 577.86 | 21.96 ±3.54 | 1.68 ±0.91 | 15.12 ±5.71 | 11.58 ±2.16 | 17.19 ±9.64 | 07.95 ±0.46 | 27.15  ±14.97 | 52.85 | 1110 | 15 |
| Nandoni | 12 | 483.78 | 23.05 ±2.23 | 1.89 ±0.77 | 16.06 ±7.97 | 12.16 ±3.11 | 21.62 ±14.54 | 07.91 ±0.60 | 33.60  ±22.82 | 71.18 | 358 | 10 |
| Basani | 12 | 466.14 | 24.31 ±3.93 | 2.21 ±0.22 | 14.85 ±10.75 | 18.41 ±3.04 | 24.01 ±16.34 | 07.61 ±0.66 | 31.29  ±22.09 | 71.18 | 565 | 12 |
| Tshikonelo | 12 | 457.22 | 23.60 ±4.07 | 2.56 ±1.39 | 15.47 ±6.22 | 22.29 ±3.77 | 22.00 ±14.16 | 07.72 ±0.26 | 34.43  ±21.97 | 72.90 | 479 | 12 |
| Tshirovha potholes | 09 | 934.02 | 18.15 ±2.83 | 1.37 ±0.66 | 14.81 ±5.61 | 11.11 ±2.41 | 14.63 ±1.02 | 06.64 ±0.55 | 22.87  ±1.64 | 91.44 | 270 | 12 |
| Tshirovha forest | 09 | 622.38 | 18.17 ±2.76 | 1.60 ±1.17 | 13.33 ±6.61 | 11.25 ±0.96 | 14.49 ±0.78 | 06.61 ±0.32 | 22.97  ±1.19 | 63.21 | 36 | 8 |
| Dzindi above Waterfall | 09 | 731.65 | 19.66 ±2.04 | 1.68 ±0.82 | 16.95 ±4.87 | 09.75 ±0.43 | 13.99 ±0.59 | 07.52 ±0.29 | 21.74  ±1.08 | 51.12 | 523 | 11 |
| Dzindi below Waterfall | 09 | 690.49 | 19.23 ±2.01 | 1.89 ±1.01 | 14.08 ±3.37 | 11.88 ±0.70 | 14.14 ±0.62 | 07.53 ±0.25 | 22.30  ±1.31 | 51.12 | 475 | 10 |
| Phiphidi | 09 | 605.32 | 20.57 ±2.76 | 1.31 ±0.60 | 13.00 ±3.80 | 12.62 ±0.48 | 13.74 ±0.92 | 07.19 ±0.14 | 21.73  ±1.03 | 60.73 | 429 | 13 |
| Lutananda Bridge | 09 | 651.19 | 19.93 ±3.17 | 2.01 ±0.95 | 14.02 ±4.16 | 09.25 ±0.96 | 14.96 ±2.64 | 07.56 ±0.20 | 22.27  ±1.75 | 27.59 | 689 | 11 |
| Hasani | 09 | 532.27 | 21.60 ±3.88 | 1.39 ±0.83 | 11.34 ±2.29 | 14.75 ±1.39 | 13.78 ±0.97 | 07.85 ±0.15 | 21.02  ±1.62 | 52.85 | 224 | 8 |
| Tshanzhe | 09 | 547.53 | 22.03 ±3.92 | 1.10 ±0.81 | 16.98 ±6.24 | 09.25 ±3.15 | 13.36 ±0.99 | 07.22 ±0.18 | 20.79  ±1.71 | 78.93 | 28 | 7 |
| Upper Lutananda | 09 | 745.57 | 19.15 ±3.72 | 1.31 ±0.69 | 14.89 ±4.21 | 05.25 ±0.43 | 13.96 ±1.21 | 07.45 ±0.55 | 25.54  ±6.98 | 27.59 | 302 | 9 |

Table S2: List of mayfly species collected over the 12 months sampling period in the Luvuvhu catchment. Indicator taxa are in bold and there abundances are labelled with an asterisk.

| **Family** | **Species** | **Abundance** |
| --- | --- | --- |
| Baetidae | *Acanthiops varius* Crass, 1947 | 123 |
|  | *Afroptilum sudafricanum* Lestage, 1924 | 1 |
|  | ***Baetis harrisoni* Barnard, 1932** | 1276* |
|  | ***Baetis* sp**. | 2734* |
|  | *Centroptiloides bifasciatum* Esben-Petersen, 1913 | 13 |
|  | *Cheleocloeon* sp. | 354 |
|  | *Cloeon* sp*.* | 12 |
|  | ***Dabulamanzia media* Crass, 1947** | 1666* |
|  | *Demoulinia crassi* Demoulin, 1971 | 7 |
|  | ***Nigrobaetis* sp.** | 847* |
|  | *Pseudocloeon glaucum* Agnew, 1961 | 272 |
|  | *Pseudocloeon* sp*.* | 13 |
|  | *Pseudoponnota maculose* Crass, 1947 | 287 |
|  | *Pseudoponnota* sp. | 4 |
|  |  | 7609 |
| Caenidae | ***Caenis* sp.** | 308* |
| Heptageniidae | *Afronurus barnardi* Schoonbee, 1968 | 914 |
| Leptophlebiidae | ***Euthraulus elegans* Barnard, 1932** | 1232* |
| Oligoneuriidae | *Elassoneuria trimeniana* McLachlan, 1868 | 52 |
| Tricorythidae | *Tricorythus discolor* Burmeister, 1839 | 926 |
|  | Grand Total | 11041 |


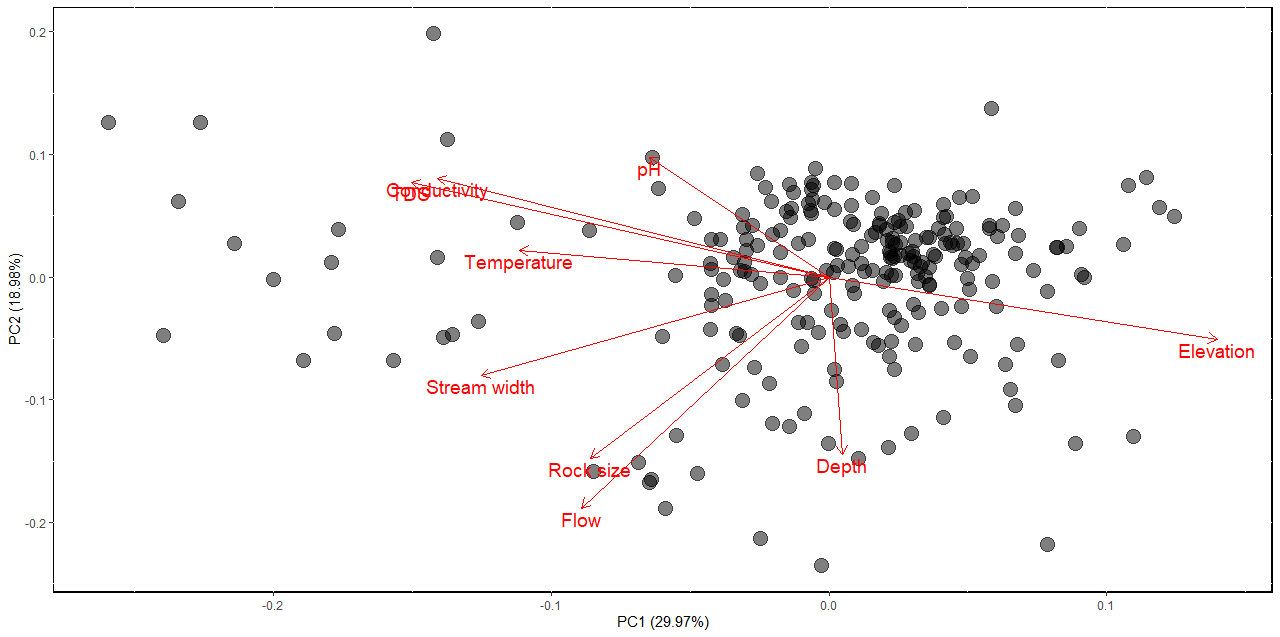


Figure S1: Principal Component Analysis of the relationship between environmental variables measured at the 23 study sites over the period of the study. Points represent surveys at study sites.
